# Sleep-Related Hypoventilation in a Patient With Ehlers-Danlos Syndrome

**DOI:** 10.7759/cureus.103980

**Published:** 2026-02-20

**Authors:** Susie X Fong, Aaron P Thomas

**Affiliations:** 1 Sleep Medicine, University of California Los Angeles David Geffen School of Medicine, Los Angeles, USA; 2 Internal Medicine-Sleep Medicine, Greater Los Angeles VA Healthcare System, Los Angeles, USA

**Keywords:** ehlers danlos syndrome, hypoventilation syndrome, muscle atonia, rapid eye movement (rem) sleep, sleep-related hypoventilation

## Abstract

Although sleep apnea is frequently observed in patients with hypermobile Ehlers-Danlos syndrome (EDS), hypoventilation is uncommon. We present a case of a patient with hypermobile EDS without sleep apnea who was found to have isolated sleep-related hypoventilation. Hypoventilation is defined by elevated PaCO_2_. It is exacerbated during sleep when ventilatory control sensitivity is diminished. Hypoventilation is further worsened during rapid eye movement (REM) sleep when accessory respiratory muscles are atonic, and breathing is driven solely by the diaphragm. This report highlights the importance of considering hypoventilation in patients with EDS, especially those with relevant risk factors.

## Introduction

Ehlers-Danlos syndrome (EDS) is a group of conditions characterized by similar symptoms, such as skin hyperextensibility, joint hypermobility, and tissue fragility, that result from defects in collagen synthesis or processing. The manifestations of this condition can involve multiple organ systems, including the vascular, gastrointestinal, hematologic, ocular, and respiratory systems. There are a total of 13 subtypes of EDS, categorized based on clinical signs and symptoms as well as inheritance patterns [1]. While most subtypes can be diagnosed using genetic markers, the most common, hypermobile EDS, lacks a known biomarker. Common and defining features of hypermobile EDS include joint hypermobility and instability, musculoskeletal manifestations, chronic fatigue and pain, exertional dyspnea, and organ prolapse [2,3].

With respect to the respiratory system, hypermobile EDS typically presents with reduced inspiratory muscle strength, dysphonia, dyspnea, hoarseness, chest wall deformities, airway collapse, weak voice, laryngospasm, subglottic stenosis, and restrictive and obstructive patterns [3,4]. Hypoventilation is not typically reported in hypermobile EDS. We suspect that this may be due to the fact that manifestations of the condition occur in the connective tissue of the respiratory system, affecting the structure and mechanics of breathing rather than ventilatory control itself. However, the combination of chest wall deformity, chest wall laxity, and respiratory muscle weakness can culminate in hypoventilation, particularly in a patient with other risk factors for this condition.

Hypoventilation refers to elevated partial pressure of carbon dioxide (PaCO_2_) and subsequent decreased partial pressure of oxygen (PaO_2_). Daytime hypoventilation is defined as PaCO_2_ 45 mmHg or greater [5]. Hypoventilation is worsened during sleep. When awake, the complex control of ventilation is dependent on central respiratory centers, central chemoreceptors, peripheral chemoreceptors, and stretch receptors. When asleep, the output of central respiratory centers is diminished, leading to increased upper airway resistance and a decrease in minute ventilation. This, in addition to reduced chemosensitivity (elevated apneic threshold) and mechanical sensitivity, results in an elevated PaCO_2_ and a correspondingly decreased PaO_2_.

During rapid eye movement (REM) sleep, hypoventilation can be further worsened due to skeletal muscle atonia, which causes reduced accessory respiratory muscle activity. Maintenance of adequate ventilation in REM thus switches to rely heavily on the diaphragm [6]. Typically, the levels of PaCO_2_ increase by 7 mmHg compared to awake levels in healthy individuals [7]. While these physiological changes do not present any issue in the general population, in a patient with a condition inhibiting or altering the above mechanisms, it can lead to hypoventilation. The definition of sleep-related hypoventilation is met when either of the following criteria is satisfied: 1) an increase in PaCO_2_ to a value greater than 55 mmHg for greater than or equal to 10 minutes, or 2) an increase of 10 mmHg or more in PaCO_2_ during sleep, compared with the awake supine value, resulting in a value exceeding 50 mmHg for 10 minutes or more [8].

This report highlights a rare example of sleep-related hypoventilation in a patient with EDS, which could potentially be an under-recognized systemic manifestation of hypermobile EDS, and hence underscores the importance of a polysomnogram with CO_2_ monitoring in symptomatic patients.

## Case presentation

The patient was a 22-year-old male with hypermobile EDS who presented with paroxysmal nocturnal dyspnea and worsening fatigue. His sleep pattern was notable for sleep-onset insomnia of 60-120 minutes, two nighttime awakenings due to night sweats/apneas, and daytime naps. He denied snoring and morning headaches. He reported episodes of choking either in the middle of the night or early morning, especially while in the supine position. His Epworth Sleepiness Scale was 2 (maximum 24), and STOP-BANG was 3 (maximum 8). His vitals were normal, and his BMI was 20. Pertinent medications include hydromorphone three times a day, oxycodone four times a day, and cyclobenzaprine nightly as needed for EDS-related pain and clonazepam as needed for anxiety. He had been on chronic opioids and benzodiazepines for at least three years before presentation. Thyroid-stimulating hormone and hemoglobin, labs typically ordered in a patient with unexplained fatigue, were within normal limits (Table 1). The patient’s serum CO_2_ was in the normal range at the time of presentation, but had been near the upper end of normal or elevated at 10-11 weeks before presentation (Table 1). Pertinent labs are listed below (Table 1).

**Table 1:** Pertinent labs This table shows the variability of serum CO_2_ levels prior to presentation

Pertinent labs		
Lab	Result	Normal range
Thyroid-stimulating hormone	2.7 mcIU/mL	0.3-4.7 mcIU/mL
Hemoglobin	14.6 g/dL	13.5-17.1 g/dL
Serum CO_2_ at 3 weeks prior to presentation	24 mmol/L	20-30 mmol/L
Serum CO_2_ at 10 weeks prior to presentation	28 mmol/L	20-30 mmol/L
Serum CO_2_ at 11 weeks prior to presentation	31 mmol/L	20-30 mmol/L

While the patient did not snore, he reported experiencing and had witnessed apneas while sleeping, daytime fatigue, and had a moderate pretest probability for sleep disordered breathing, which prompted an in-lab polysomnogram (PSG). His chronic use of opioids was a risk factor for hypoventilation, leading to the addition of transcutaneous carbon dioxide (TcCO_2_) monitoring. Of note, TcCO_2 _monitoring includes the use of a skin sensor, which provides a non-invasive but limited way to trend CO_2_ levels in situations that do not require more definitive values that an arterial blood gas is able to provide. The patient underwent an in-lab PSG with TcCO_2_ monitoring, with the following results (Table 2).

**Table 2:** Polysomnogram This study demonstrates the absence of sleep disordered breathing with the presence of hypoventilation AHI: apnea hypopnea index; RDI: respiratory disturbance index; TcCO_2_: transcutaneous carbon dioxide; NREM: non-rapid eye movement; REM: rapid eye movement; TST: total sleep time

Polysomnogram		
Indices	Result	Normal range
Total recording time	7.1 hours	N/A
Total sleep time	6.9 hours	N/A
AHI	1/hour	<5/hour
RDI	3/hour	<5/hour
Central Apnea Index	0.14/hour	<5/hour
Oxygen saturation nadir	90%	>88%
Oxygen saturation baseline	97%	95%-100%
Oxygen saturation <88%	0 minute	<5 minutes
Periodic limb movement index	0/hour	<15/hour
TcCO_2_ baseline	51-53 mmHg	45-55 mmHg
TcCO_2_ >55 mmHg	13.7 minutes	<10 minutes
TcCO_2_ min and max in wake	45, 57 mmHg	45-55 mmHg
TcCO_2_ min and max in NREM	42, 57 mmHg	45-55 mmHg
TcCO_2_ min and max in REM	48, 53 mmHg	45-55 mmHg
Stage N1	10% of TST	5-15%
Stage N2	72% of TST	45-80%
Stage N3	0% of TST	0-15%
Stage R	18% of TST	12-20%

The patient’s PSG did not demonstrate sleep disordered breathing, but the presence of hypoventilation with TcCO_2_ greater than 55 mmHg for 13.7 minutes (Table 2). This result met the criteria for sleep-related hypoventilation with arterial PCO_2 _increase greater than 55 mmHg, for greater than or equal to 10 minutes. The PSG also revealed normal distribution of sleep stages except for the absence of stage N3 sleep, which can, in some cases, be secondary to sleeping in a foreign environment. Of note, the patient was only supine during this PSG. There was no relationship between TcCO_2_ levels and arousals. The patient subsequently underwent bilevel positive airway pressure (BPAP) titration with the following results (Table 3).

**Table 3:** Polysomnogram titration This study demonstrates transcutaneous carbon dioxide levels <55 mmHg with BPAP therapy BPAP: bilevel positive airway pressure; AHI: apnea hypopnea index; RDI: respiratory disturbance index; TcCO_2_: transcutaneous carbon dioxide; NREM: non-rapid eye movement; REM: rapid eye movement; TST: total sleep time

Polysomnogram titration		
Indices	Result	Normal range
Total recording time	7.4 hours	N/A
Total sleep time	6.9 hours	N/A
AHI	0.6/hour	<5/hour
Oxygen saturation nadir	94%	>88%
Oxygen saturation baseline	99%	95-100%
Oxygen saturation <88%	0 minute	<5 minutes
Periodic limb movement index	0/hour	<15/hour
TcCO_2_ baseline	45 mmHg	45-55 mmHg
TcCO_2_>55 mmHg	0 minute	<10 minutes
TcCO_2_ min and max in wake	32, 47 mmHg	45-55 mmHg
TcCO_2_ min and max in NREM	36, 48 mmHg	45-55 mmHg
TcCO_2_ min and max in REM	43, 47 mmHg	45-55 mmHg
Stage N1	10% of TST	5-15%
Stage N2	66% of TST	45-80%
Stage N3	5% of TST	0-15%
Stage R	19% of TST	12-20%

A BPAP setting of 9/5 cm H_2_O was found to adequately maintain TcCO_2_ below hypoventilation levels (Table 3). There was no relationship between TcCO_2_ levels and position, nor with arousals during the titration study.

The patient was prescribed BPAP 9/5 cm H_2_O. The patient’s serum CO_2_ after one to two months of consistent BPAP usage ranged from 21 to 28 mmol/L (Table 4). 

**Table 4:** Serum carbon dioxide after BPAP therapy This table demonstrates the reduction in serum carbon dioxide after starting BPAP therapy BPAP: bilevel positive airway pressure

Serum CO_2_ after BPAP therapy		
Weeks after starting therapy	Serum CO_2_	Normal range
2	27 mmol/L	20-30 mmol/L
7	21 mmol/L	20-30 mmol/L
8	28, 24, 26, mmol/L	20-30 mmol/L

## Discussion

Despite having a moderate pre-test probability for obstructive sleep apnea, the patient’s PSG revealed a normal AHI of 1/hr and RDI of 3/hr (Table 2). The prominent finding was sleep-related hypoventilation diagnosed by transcutaneous carbon dioxide monitoring, which revealed baseline TcCO_2_ of 51-53 mmHg and TcCO_2_ 55 mmHg or higher for 13.7 minutes (Table 2). In a subsequent polysomnogram titration, a BPAP setting of 9/5 cm H_2_O kept TcCO_2_ levels below 55 mm Hg with a baseline at 45 mmHg (Table 3). The patient had reported improved breathing during the titration. In both baseline and titration polysomnograms, there was no evidence of nocturnal hypoxemia, with time oxygen saturation less than 88% for 0 minute (Tables 2, 3). The pattern of elevated TcCO_2_ readings across sleep stages did not show worsening during REM sleep as expected. Instead, the maximum TcCO_2_ in both baseline and titration polysomnograms occurred in either NREM or wake stages (Tables 2, 3). One hypothetical reason could be that the patient already has severe baseline respiratory muscle weakness, such that during REM sleep, the additional muscle paralysis does not worsen the situation beyond baseline. As a result, there was not much differentiation in TcCO_2_ levels when analyzed based on sleep stages.

The patient was prescribed BPAP 9/5 cm H_2_O. On follow-up, after using BPAP consistently for more than 4 hours nightly for at least 95% of the time, he had reported improved breathing during sleep, absence of apneas, but persistent excessive daytime sleepiness. Quantitatively, his serum CO_2_ was lower in comparison to values before presentation. Before presentation, his serum CO_2 _ranged from 24-31 mmol/L (Table 1), and after using BPAP consistently, his values ranged from 21-28 mmol/L (Table 4). While his CO_2_ values overall were lower, he continued to have persistent daytime sleepiness, which was likely multifactorial, from his EDS, opioid use, and other multiple comorbidities, which were not brought out in this case for simplicity’s sake; these other comorbidities do not contribute to hypoventilation. Of note, he did report daytime shortness of breath both at rest and with exertion, but had mixed results when he wore the BPAP in an attempt to help those symptoms.

The finding of isolated sleep-related hypoventilation in the absence of sleep disordered breathing, nocturnal hypoxemia, was intriguing and prompted further investigation. The patient was referred to pulmonology for further work-up of his sleep-related hypoventilation. Common causes of hypoventilation include: 1) neuromuscular disease (e.g., Duchenne muscular dystrophy, spinal muscular atrophy, amyotrophic lateral sclerosis), 2) restrictive chest wall disorders (e.g., obesity hypoventilation syndrome, kyphoscoliosis), 3) obstructive lung disease (e.g., chronic obstructive pulmonary disease, cystic fibrosis), and 4) central hypoventilation (e.g., brainstem injury, congenital central hypoventilation syndrome, opioids) [4,6]. These conditions elevate PaCO_2_ either by way of decreased respiratory muscle response, impaired ventilatory drive, or restricted chest wall mechanics.

The patient subsequently had a pulmonary function test revealing normal spirometry, but a suggestion of diaphragmatic weakness with a decrease in supine forced vital capacity greater than 10%. There was also a presence of severely reduced maximal inspiratory pressure (>-40 cm H_2_O) and maximal expiratory pressure (<40 cm H_2_O), suggesting respiratory muscle weakness. He subsequently had an ultrasound sniff test showing bilateral normal diaphragm motion. He was then referred to Neurology for further evaluation. Unfortunately, the patient died before completion of the workup, unrelated to hypoventilation. While the patient did not have typical signs or symptoms of hypoventilation, such as increased body mass index for obesity hypoventilation syndrome or a chronic obstructive pulmonary disease diagnosis, our patient had other risk factors that could have easily been overlooked. These include opioid use, severe respiratory muscle weakness, and possible scoliosis. 

The patient had been on chronic opioids for at least 3 years. Opioids depress the central respiratory center, which reduces ventilatory response, increases the arousal threshold, and further relaxes the upper airway muscle tone, all of which can contribute to hypoventilation [9]. Chronic pain is commonly reported in patients with hypermobile EDS, most often resulting from hypermobility, subluxations/dislocations, and myalgias. As connective tissue is present throughout the body, pain may involve virtually any organ system [1,10]. Management of these symptoms commonly involves opioid therapy, which contributes to an indirect association between EDS and hypoventilation. 

Although nighttime hypoventilation has not been specifically reported in hypermobile EDS, it is described in two rare subtypes: spondylodysplastic EDS due to beta-3GalT6-deficiency and myopathic EDS [11]. In both these subtypes, severe muscle atonia is a common characteristic. It is plausible that a similar degree of muscle weakness could have contributed to hypoventilation in this case. Notably, the patient’s maximal inspiratory and maximal expiratory pressures were both severely reduced, supporting respiratory muscle weakness. Additionally, the patient reported a history of scoliosis, which, although not confirmed, can restrict chest wall motion. A restrictive chest wall disorder contributes to hypoventilation by way of reduced tidal volumes and minute ventilation [5]. 

In this case, significant muscle atonia and opioids, combined with the reduced chemosensitivity and mechanical sensitivity in sleep, were the main contributors to hypoventilation, with scoliosis as a possible contributor. As further neurological workup could not be completed, a neuromuscular process cannot be fully ruled out.

## Conclusions

Hypoventilation in EDS is uncommon. However, it may occur in EDS patients with the appropriate combination of risk factors. In our patient, these included opioid use, respiratory muscle weakness, and possible chest wall deformity. An in-lab PSG with CO2 monitoring is a key component of the workup to evaluate for sleep apnea and hypoventilation. This report emphasizes the importance of considering hypoventilation as part of the differential when evaluating a patient with EDS who presents with nocturnal symptoms and daytime fatigue.
